# The Influence of Palmatine Isolated from *Berberis sibirica Radix* on Pentylenetetrazole-Induced Seizures in Zebrafish

**DOI:** 10.3390/cells9051233

**Published:** 2020-05-16

**Authors:** Kinga Gawel, Wirginia Kukula-Koch, Dorota Nieoczym, Katarzyna Stepnik, Wietske van der Ent, Nancy Saana Banono, Dominik Tarabasz, Waldemar A. Turski, Camila V. Esguerra

**Affiliations:** 1 Chemical Neuroscience Group, Faculty of Medicine, Centre for Molecular Medicine Norway, University of Oslo, Gaustadalléen 21, 0349 Oslo, Norway; w.v.d.ent@ncmm.uio.no (W.v.d.E.); n.s.banono@ncmm.uio.no (N.S.B.); c.v.esguerra@ncmm.uio.no (C.V.E.); 2 Department of Experimental and Clinical Pharmacology, Medical University of Lublin, Jaczewskiego Str. 8b, 20-090 Lublin, Poland; waldemar.turski@umlub.pl; 3 Chair and Department of Pharmacognosy, Medical University of Lublin, 1, Chodzki Str. 1, 20-093 Lublin, Poland; virginia.kukula@gmail.com (W.K.-K.); dominikanes@o2.pl (D.T.); 4 Department of Animal Physiology and Pharmacology, Institute of Biology and Biochemistry, Faculty of Biology and Biotechnology, Maria Curie-Skłodowska University, Akademicka Str. 19, 20-033 Lublin, Poland; dorota.nieoczym@poczta.umcs.lublin.pl; 5 Department of Physical Chemistry, Institute of Chemical Sciences, Faculty of Chemistry, Maria Curie-Skłodowska University, Pl. M. Curie-Skłodowskiej 3/243, 20-031 Lublin, Poland; katarzyna.stepnik@poczta.umcs.lublin.pl

**Keywords:** *Berberis sibirica radix*, palmatine, berberine, isolation, zebrafish, pentylenetetrazole, seizures, locomotor activity, EEG, anticonvulsant activity

## Abstract

Palmatine (PALM) and berberine (BERB) are widely identified isoquinoline alkaloids among the representatives of the Berberidaceae botanical family. The antiseizure activity of BERB was shown previously in experimental epilepsy models. We assessed the effect of PALM in a pentylenetetrazole (PTZ)-induced seizure assay in zebrafish, with BERB as an active reference compound. Both alkaloids were isolated from the methanolic root extract of *Berberis sibirica* by counter-current chromatography, and their ability to cross the blood–brain barrier was determined via quantitative structure–activity relationship assay. PALM exerted antiseizure activity, as confirmed by electroencephalographic analysis, and decreased *c-fos* and *bdnf* levels in PTZ-treated larvae. In a behavioral assay, PALM dose-dependently decreased PTZ-induced hyperlocomotion. The combination of PALM and BERB in ED_16_ doses revealed hyperadditive activity towards PTZ-induced hyperlocomotion. Notably, we have indicated that both alkaloids may exert their anticonvulsant activity through different mechanisms of action. Additionally, the combination of both alkaloids in a 1:2.17 ratio (PALM: BERB) mimicked the activity of the pure extract, which indicates that these two active compounds are responsible for its anticonvulsive activity. In conclusion, our study reveals for the first time the anticonvulsant activity of PALM and suggests the combination of PALM and BERB may have higher therapeutic value than separate usage of these compounds.

## 1. Introduction

Epilepsy is a common neurological disorder that affects approximately 65 million people worldwide, regardless of their age, sex, descent, or economic status [1]. It is manifested by repetitive seizures caused by abnormal electrical activity of neurons in the central nervous system (CNS). Currently available antiseizure drugs (ASDs), which are the main treatment for epilepsy, suppress symptoms of the disease (convulsions), but do not alleviate the process of epileptogenesis. Moreover, 30–40% of epileptic patients on antiseizure medication do not experience satisfactory improvement, i.e., no reduction of the frequency and/or severity of seizures [2]. Adverse effects of ASDs are an additional problem in epilepsy treatment and are presumed to be the main reason for discontinuation of treatment in about 25% of epileptic patients [3]. Therefore, there is an urgent need to search for new, more effective and safer antiseizure medications. In this context, plant-derived compounds might be an attractive alternative to existing ASDs. Moreover, the search for efficient anticonvulsive plant-derived compounds may help to uncover new mechanisms of anticonvulsant action and deliver currently unconsidered chemical structures for further development.

Zebrafish (*Danio rerio*) is now recognized as a useful model organism in biomedical research for CNS disorders, such as schizophrenia, anxiety, addiction, or disturbances of learning and memory. With regard to epilepsy, a number of genetic disease models recapitulating human phenotypes have been described to date [4,5,6,7,8]. Pharmacological models are widely used for high-throughput screening of new compounds for the treatment of seizures, the most commonly used model being the acute pentylenetetrazole (PTZ)-induced seizure assay, in which tonic-clonic-like seizures are correlated with hyperlocomotion in larval zebrafish [9,10]. The behavioral manifestation of seizures corresponds well with the high number of epileptiform-like discharges observed at the electroencephalographic (EEG) level, and compounds with anticonvulsant activity prevent PTZ-induced hyperlocomotion. Using the PTZ assay, different marine-derived, plant-derived, and synthetic compounds have been found to possess anticonvulsant activity [11,12,13,14,15]. The PTZ assay has been shown to correlate well with equivalent PTZ model in mice [11,12,15].

**Figure 1:**
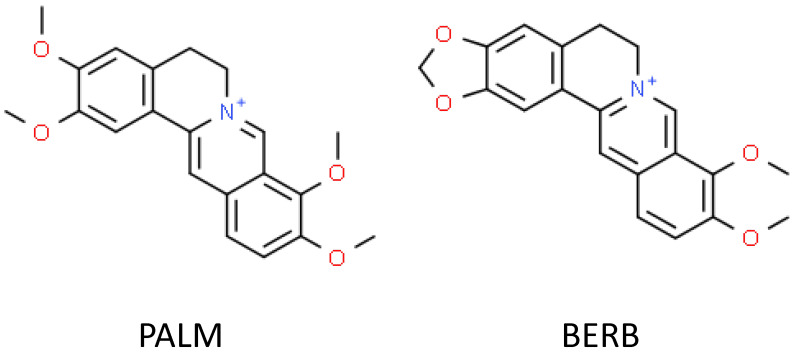
The chemical structure of palmatine (PALM) and berberine (BERB).

The protoberberine alkaloid palmatine (PALM) is a quaternary salt, a derivative of isoquinoline substituted with four methoxyl groups (Figure 1), and is widely identified among representatives of the Berberidaceae, Papaveraceae, Ranunculaceae, Menispermaceae, and Rutaceae botanical families. Traditionally, it is used to relieve hypertension, inflammation, dysentery, or diseases of the liver. Recent studies on its pharmacological applications confirm its significant potential in the treatment of CNS-related diseases, obesity, and cancer [16,17].

In this study, PALM was selected based on the previously published scientific data that confirmed its ability to cross the blood–brain barrier (BBB) [16] and the results of mathematical modeling towards the BBB permeation performed in this paper. Quantitative structure–activity relationship (QSAR) studies were conducted in order to construct new assessment models of PALM pharmacological behavior, such as brain pharmacokinetic descriptors, namely, the blood–brain barrier penetration descriptor (logBB), the permeability-surface area product (logPS), the brain-plasma equilibration rate (logPS*fu,brain) and brain fractions unbound in plasma and brain.

We aimed for the first time to assess the influence of PALM, isolated from the methanolic extract of *Berberis sibirica* root, on the PTZ-induced seizure assay in larval zebrafish. We used a novel separation technique, centrifugal partition chromatography (CPC, also known as hydrostatic counter-current chromatography), to isolate PALM from the complex extract of *Berberis sibirica* roots. This isolation method was selected based on its high recovery efficiency of natural products from total extracts due to the lack of solid adsorbent and the possibility to perform large-scale separation suitable for in vivo studies. To confirm the anticonvulsant properties of PALM in the zebrafish larvae at the molecular level, expression of two seizure-related genes, i.e., *bdnf* and *c-fos*, was determined. Both BDNF and c-fos are recognized as markers of neuronal activity that participate in the epileptogenesis, and their upregulation after seizures was found both in the rodent [18,19] and zebrafish [9,20,21,22] seizure models.

We also compared the anticonvulsant activity of PALM with the activity of berberine (BERB) (Figure 1), another widely distributed and well-studied protoberberine alkaloid, which is known to exert antiseizure activity both in rodent and zebrafish [22,23,24] models and can be used as an active reference compound. Finally, we evaluated the anticonvulsant activity of the combination of different doses of PALM and BERB.

## 2. Materials and Methods

### 2.1. Quantitative Structure–Activity Relationship (QSAR) Studies

Within *in silico* studies, ACD/Percepta software (version 2012, Advanced Chemistry Development, Inc., Toronto, ON, Canada) was used. QSAR analysis was made using the Minitab 18 Statistical Software (Minitab Inc., State College, PA, USA).

### 2.2. Extraction

The plant material used in the study (*Berberis sibirica* Pall.) was collected and authenticated by dr. Otgonbataar Urjin from the Mongolian National University of Medical Sciences in Ulanbataar, Mongolia. The area of collection was the Bayan Province of Mongolia (July 2010). The cut, air-dried and ground up root was labeled with voucher specimen number WK2010007. Its sample is now deposited in the Chair and Department of Pharmacognosy, at the Medical University of Lublin, Poland by Wirginia Kukula-Koch. Two 20 g portions of the powdered plant material were extracted with methanol by a pressurized liquid extractor (also known as an accelerated solvent extractor–ASE) (ASE 100, Dionex, Sunnyvale, CA, USA) using the following conditions: static time: 5 min, number of cycles: five, temperature: 90 °C, purge time: 120 s, purge volume: 60%, stainless steel cell size: 66 mL. The pressure was maintained at ca. 96 bar during the whole extraction process. The obtained extracts were combined and evaporated until dryness under decreased pressure conditions in a rotary evaporator at 45 °C. The dried residue constituted 17% of the powdered root.

### 2.3. Chromatographic Analysis of the Extract by HPLC-ESI-Q-TOF-MS

High-performance liquid chromatography coupled with mass spectrometry (HPLC-MS) was used for the qualitative composition assessment of the obtained extract and fractions from the counter-current separation. An HPLC chromatograph (HP 1200 series, Agilent Technologies, Santa Clara, CA, USA) equipped in a degasser, a binary pump, an autosampler, a column thermostat and a UV-PDA detector coupled with a Q-TOF mass spectrometer with an electrospray ionization (ESI) source (6500 Series, Agilent Technologies, Santa Clara, CA, USA) was used in the study. The instrument was calibrated right before the injections, and the operating parameters were initially optimized in both negative and positive ionization mode. The following conditions were applied to all samples: gas and sheath gas flows—12 L/min, gas temperature: 350 °C, sheath gas temperature: 325 °C, fragmentor voltage: 120 V, skimmer voltage: 65 V, nebulizer voltage: 30 psig, capillary voltage: 3500 V, collision energy: 20 and 40 V. The spectra were collected within the *m/z* range of 100–1000. The MS/MS spectra of alkaloids were recorded out of the two most intensive signals in a scan. Internal calibrants were used throughout the analysis to sustain the accuracy of mass measurements. In the constructed method, after the collection of one spectrum of a given *m/z* value, the selected peaks were excluded from fragmentation for 0.3 min to trace the fragmentation spectra of the weaker signals. The HPLC method with Zorbax Eclipse Plus stable bond RP-18 chromatographic column (150 × 2.1 mm, dp = 3.5 µm, Agilent Technologies, Santa Clara, CA, USA) run under the following gradient of solvent A (0.1% formic acid) and solvent B (acetonitrile with 0.1% formic acid): 10% of B in A at 0 min, 40% of B in A at 10 min, isocratic run at 40% of B in A for the following 2 min, 95% B in A at 21 min. The post-run was set at 10 min, the flow rate at 0.2 mL/min, the temperature at 25 °C and the detection wavelengths at 254, 280, 290, 365 nm. Mass Hunter Workstation and Qualitative Navigator B.08.00 software was used to record and handle the obtained data, respectively.

### 2.4. Centrifugal Partition Chromatography (CPC)-Based Fractionation of the Extract

PALM was isolated from the total extract of *Berberis sibirica* on a 250-mL column using an Armen Spot CPC-250-L instrument, that was equipped with a rotor, external UV detector (Flesh06S DAD 600) and fraction collector (LS-5600) (Armen Instrument, Saint Ave, France). From a variety of biphasic solvent systems tested to select the most optimal separation conditions, the mixture of ethyl acetate: 1-butanol: ethanol: water (3:1:1:6 *v*/*v*/*v*) was used after Tang et al. [25] with several modifications to adjust the methodology to different separation conditions, that provides CPC chromatograph in relation to the one used by Tang HPCCC instrument. First, the partition coefficient values (*k*) were determined for each alkaloid from the extract. For this purpose, a test tube assay was performed. Five mL of the biphasic solvent system was prepared, and ca. 20 mg of dried extract was dissolved in this biphasic mixture. Then, 1 mL portions of the upper and the lower phases were separately transferred to two separate autosampler vials. The content was dried out on a rotary evaporator (Eppendorf Concentrator Plus, Eppendorf, Hamburg, Germany), the residues were redissolved in 1 mL of methanol and subjected to HPLC-MS analysis. The *k* values were calculated from each obtained chromatogram, separately for each compound, by dividing its peak area in the lower phase by the corresponding peak area in the upper phase. Based on the obtained data and the differences in the *k* values between the compounds of interest, the ascending operation mode was selected for the analysis and the following chromatographic conditions were applied: flow rate: 6 mL/min, rotations of the column: 1800 RPM, detection wavelength: 280 and 320 nm, elution time: 30 min, extrusion time: 50 min. During the separation process, the entire volume of the column was first filled with the stationary phase, and the mobile phase was pumped together with an injection of the analyzed sample (200 mg). After the separation, the obtained fractions were transferred to autosampler vials through a nylon syringe filter, evaporated to dryness, redissolved in methanol, and subjected to the quantitative analysis by HPLC-MS.

BERB was isolated from the aerial parts of the plant according to the previously published protocol [26].

### 2.5. Drugs and Reagents

PTZ was purchased from Sanofi Aventis (Paris, France). All solvents used for the HPLC-MS analysis (water, acetonitrile, formic acid of LC-MS purity grade) were purchased from Avantor Performance Materials (Gliwice, Poland). Reagent grade solvents for extraction and counter-current separation were manufactured by Avantor Performance Materials (Gliwice, Poland).

### 2.6. Zebrafish Maintenance and Breeding

Adult zebrafish of wild type AB strain (Zebrafish International Resource Center, Eugene, OR, USA) were kept at 28.5 °C on a 14-h light/10-h dark cycle under standard housing protocols. Embryos resulting from natural spawning were raised in E3 embryo medium (1.5 mM HEPES, pH 7.6, 17.4 mM NaCl, 0.21 mM KCl, 0.12 mM MgSO_4_, and 0.18 mM Ca(NO_3_)_2_), on a 14-h light/10-h dark cycle and at 28.5 °C. Larvae used for all assays were up to seven days postfertilization (dpf). All animal experiments received approval from the experimental animal administration’s supervisory and application system of the Norwegian Food Safety Authority (“Forsøksdyrforvatningentilsyns-ogsøknadssystem”; FOTS 18/106800-1).

### 2.7. EEG Analysis in Zebrafish

In order to identify seizure-like discharges in zebrafish larvae, the recordings of EEG were carried out in accordance with the previously described method by Afrikanova et al. [10]. A glass electrode (resistance 1–5 MΩ) filled with artificial cerebrospinal fluid (2 mM KCl, 124 mM NaCl, 2 Mm CaCl_2_, 2 mM MgSO_4_, 1.25 26 mM NaHCO_3_, mM KH_2_PO_4_, 10 mM glucose) was inserted in the optic tecta of 7 dpf zebrafish larvae. Individual larva were embedded in a thin layer of 2% low-melting-point agarose to restrain them during a 20 min recording period. Analysis of the data was performed via a combination of the Clampfit software version 10.2 (Molecular Devices Corporation, San Jose, CA, USA) and a custom-written R script. 

### 2.8. Quantitative Real-Time Polymerase Chain Reaction (qRT-PCR)

Larvae (7 dpf, *n* = 10/sample, *n* = 4/group) were pretreated with 450 µM PALM or vehicle for 24 h with subsequent 45 min long exposure to 20 mM PTZ. Next, zebrafish heads were collected in a pool of 10 per sample. TRIZOL was used for the purification of RNA and SuperScript™ IV First-Strand Synthesis System (Invitrogen, Carlsbad, CA, USA) was used in cDNA synthesis. To amplify the cDNA, PowerUp™ SYBR™ Green Master Mix (Applied Biosystems, Foster City, CA, USA) was used in accordance with the manufacturer’s instructions. The 2^−∆∆t^ method was used to compute relative enrichment [27]. The levels of expression of *c-fos* and *bdnf* (genes of interest) were normalized against glyceraldehyde 3-phosphate dehydrogenase (*gapdh)* which acted as a housekeeping gene. Primer sequences:

gapdh_f_5′GTGGAGTCTACTGGTGTCTTC3′,
gapdh_r_5′GTGCAGGAGGCATTGCTTACA3′;
c-fos_f_5′CCGATACACTGCAAGCTGA 3′,
c-fos_r_ 5′TGCGGCGAGGATGAACTCTA3′;
bdnf_f_5′AGCTGAAGAGACAACTTGC3′,
bdnf_r_5′CCATAGTAACGAACAGGAT3′.

Experiments were twice replicated and data were pooled together.

### 2.9. Behavioral Analysis of Anticonvulsant Activity in Zebrafish

Larval zebrafish locomotor activity was measured as previously described by Afrikanova et al. [10]. Larvae (6 dpf) were put in a 48-well plate (individual larva per well). Each well contained either 200 µL of vehicle, BERB or PALM solutions. Larvae were subsequently incubated at 28.5 °C for 20 h. Afterward, 100 µL of vehicle or PTZ (60 mM) was added to each well. In a space of 5 min, plates were placed in the ZebraBox, an automated tracking device (Viewpoint, Lyon, France). The distance travelled by each larva in millimeters (mm) was recorded during a 30 min tracking duration, with an integration interval of 5 min. All locomotor tracking were implemented at the same period of the day. Data were pooled together from three independent experiments.

### 2.10. Statistical Analysis

Statistical analysis was conducted with the aid of GraphPad Prism 7.04 version (San Diego, CA, USA). Data are presented as a mean ± SEM (standard error of the mean). The ED_16_ value (16% of anticonvulsive dose), ED_50_ value (50% of anticonvulsive dose), and its 95% confidence limits were calculated by a computer probit-log linear regression analysis according to Litchfield and Wilcoxon [28]. The data were analyzed by one-way or two-way analysis of variance (ANOVA) with repeated measures, followed by Tukey’s or Bonferroni’s *post-hoc* test, respectively. Student’s *t*-test was used if applicable. Differences were considered statistically significant if the *p*-value was less than 0.05.

## 3. Results

### 3.1. Quantitative Structure–Activity Relationship (QSAR) Studies for the BBB Permeation of PALM

To estimate the BBB penetration of PALM, an entirely new QSAR model has been established. For this purpose, the BBB penetration descriptor expressed as the logarithm of the ratio of a compound concentration in the brain to the concentration in the blood (logBB) [29] values were taken into account. The dataset used here includes 13 structurally similar compounds, which belong to the alkaloids, with reported activity on the CNS. The new established QSAR model combines the experimentally determined logBB values (Table 1) taken from the literature [30,31,32,33,34,35,36,37] with chosen physicochemical descriptors, namely, the hydrogen bond acidity A value, the lipophilic logP_o/w_ value, and the molecular weight (MW) (Table 1). The hydrogen bond acidity values were calculated based on the Linear Free Energy Relationships (LFER) of the Abraham model [38]. Our model was intended in such a way that the tested PALM was external for the model, meaning that it was not used to develop the model. In the process of constructing our model, we used the Hansch approach, according to which there is a relationship between molecule transport across biological barriers, including the BBB, and the steric, lipophilic, and electronic characteristics of a molecule [39,40].

logBB = 0.403−2.907A−0.398 logP_o/w_ + 0.00376 MW *n* = 13, R^2^ = 73.59%, SE = 0.276 (1)

In order to confirm the predictability of the model (Equation (1)), the analysis of variance was carried out (Table 2).

The precision of the estimation of an unknown value of a given coefficient (logBB value) can be estimated using the standard error of a coefficient (SE coefficient). The standard error is observed to have a lower value in the case of MW than for the other considered descriptors. This may suggest that the logBB value of the tested alkaloids, including PALM, is better characterized by MW than by other parameters. 

The *p*-Value, being the probability that measures the evidence against the null hypothesis, for all the tested parameters was smaller than the significance level. This indicates that there is not a statistically significant association between the response variable and the logBB values. Moreover, taking into account the obtained Variance Inflation Factor (VIF) values, it can be stated that all the values are lower than 5, which means that multicollinearity does not exist in the context of the correlation between predictors. In addition, all the variables are associated with the calculated logBB values, which can be proved by the obtained F-Values higher than 1 in each case. In summary, the analysis of variance shows that the model corresponds well to real data, thus its predictability and applicability for the studied alkaloids, including PALM, can be confirmed.

Based on the newly established model, the logBB value for PALM has been calculated. The obtained logBB value is equal to −0.18. Moreover, logBB value was calculated using computational *in silico* approach (ACD/Percepta software). This value was equal to −0.26. Analyzing both logBB values (higher than −1), it can be assumed that PALM can cross the BBB. Moreover, pharmacokinetic parameters calculated *in silico* provided sufficient data to consider the application of PALM in CNS disorders.

### 3.2. Isolation of PALM and BERB from Plant Material

The methanolic extract from Siberian barberry was found to contain a variety of isoquinoline alkaloids in the HPLC-ESI-Q-TOF-MS tests. Among them, several representatives of protoberberines, aporphines, bisbenzylisoquinolines, or benzylisoquinolines were tentatively identified, with the highest concentration attributed to BERB, PALM, magnoflorine, and jatrorhizine.

To achieve an efficient separation of these metabolites, which are closely related to one another from a chemical point of view, CPC-based isolation was proposed. This technique offers high selectivity and lack of solid support that helped an efficient separation.

The determination of partition coefficient values (*k*) for each compound in the extract was performed to select a proper biphasic solvent system for the isolation. Large differences between *k* values calculated for the major alkaloids, namely magnoflorine: 10, jatrorhizine: 5.4, PALM: 6.3, BERB: 2.7, confirmed a good selectivity of the mixture of ethyl acetate: 1-butanol: ethanol: water (3:1:1:6 *v*/*v*/*v*). Importantly, the *k* value of BERB in the proposed solvent system had a markedly lower value than the other compounds, and it could be expected that under these conditions it could be eluted separately from PALM.

**Figure 2:**
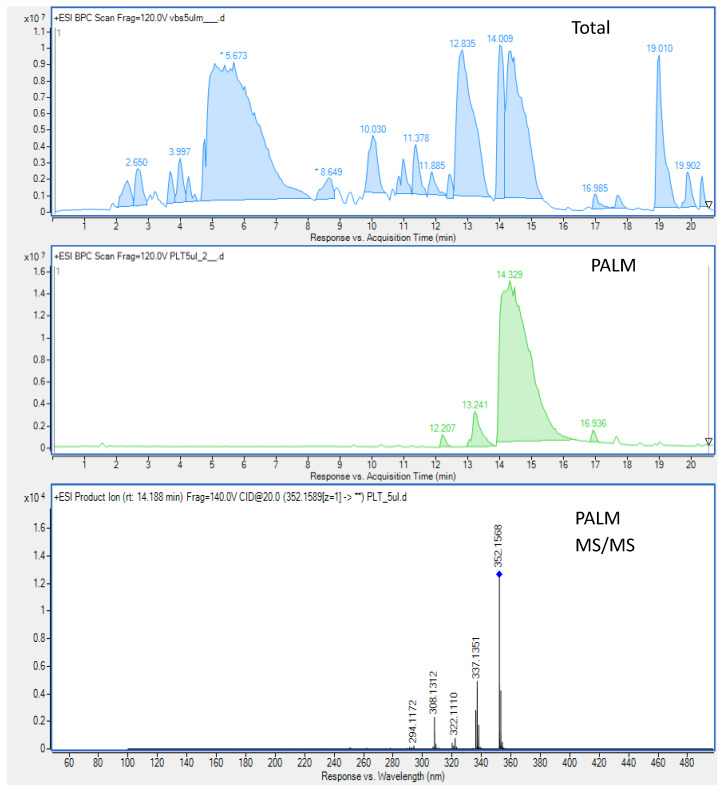
The composition of the total extract, purified palmatine (PALM), and its MS/MS spectrum (PALM MS/MS).

CPC was found to be efficient enough to purify PALM from the total methanol extract of *Berberis sibirica* Pall. (Figure 2). PALM was obtained after 50 min, in the extrusion mode of analysis at a purity exceeding 93%. The fragmentation pattern of the alkaloid was consistent with the literature data. The MS/MS spectrum of PALM showed clear *m/z* signals of 337, 322, 308, and 294 next to the parent ion [M + H]^+^ of 352, which is in accordance with previous findings [41,42] and describe the sequence of methyl and hydroxyl groups’ detachment out of the parent ion.

### 3.3. The Influence of PALM or BERB on PTZ-Induced EEG Discharges in Zebrafish

**Figure 3:**
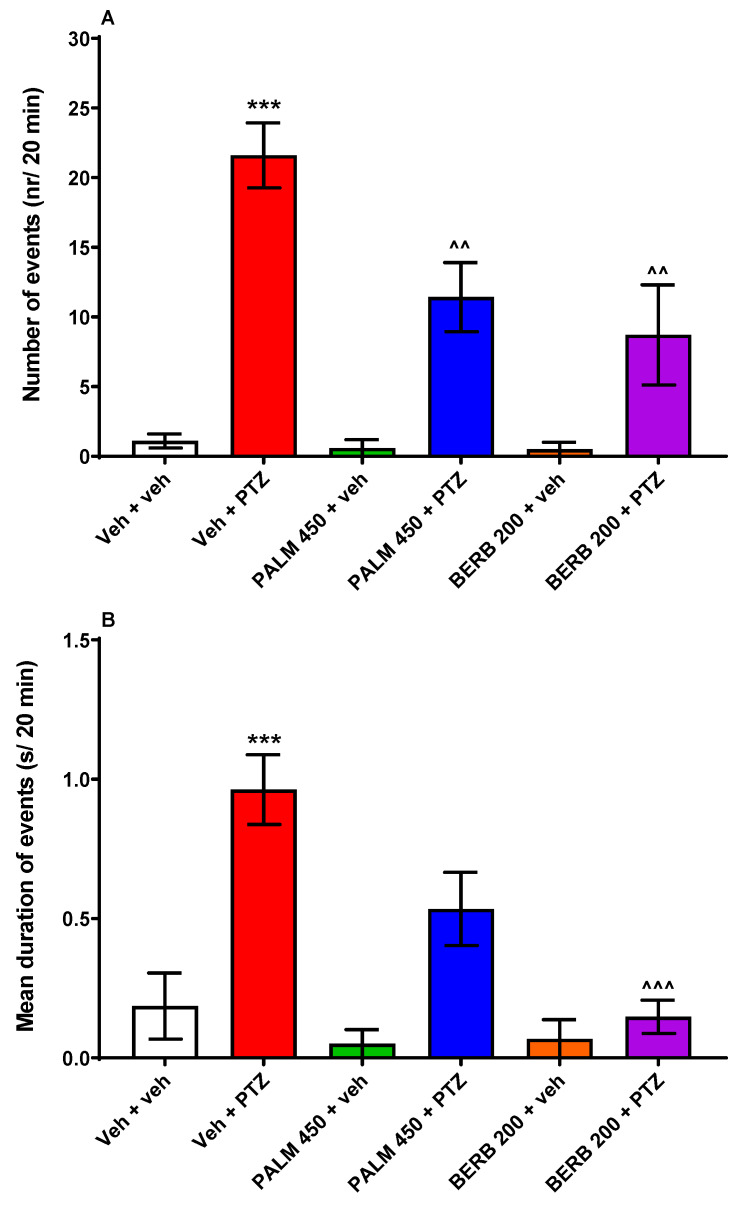
Electroencephalographic (EEG) recordings obtained from the optic tectum of 7 dpf zebrafish larvae pre-incubated for 24 h with PALM (450 µM) or BERB (200 µM). Data are shown as (**A**) number of events (nr/20 min) and (**B**) mean duration of events (s/20 min). Data were analyzed using one-way ANOVA followed by the Tukey’s *post-hoc* test. Data are depicted as mean ± SEM. Veh + veh (*n* = 10), Veh + PTZ (*n* = 15), PALM 450 + veh (*n* = 5), PALM 450 + PTZ (*n* = 14), BERB 200 + veh (*n* = 4), BERB 200 + PTZ (*n* = 8). *** *p* < 0.001 vs. Veh + veh; ^^^ *p* < 0.001, ^^ *p* < 0.01 vs. Veh + PTZ. BERB—berberine, PALM—palmatine, PTZ—pentylenetetrazole, Veh—vehicle.

The EEG recordings were obtained from the optic tectum of 7 dpf larval zebrafish. One-way ANOVA revealed statistically significant differences between the tested groups of zebrafish larvae regarding the number (F (5, 49) = 12.62, *p* < 0.001; *n* = 4–15/group; Figure 3A) and mean duration of events (F (5, 49)  =  8.57, *p* < 0.001; *n* = 4–15/group; Figure 3B). PALM (450 µM) and BERB (200 µM) significantly reduced number of epileptiform-like discharges in comparison to the PTZ-treated group (*p* < 0.01). Although PALM (450 µM) had a tendency to decrease the mean duration of events, the results did not reach statistical significance (*p* > 0.05). On the other hand, BERB (200 µM) substantially decreased the mean duration of events in PTZ-treated fish (*p* < 0.001). Neither PALM nor BERB affected the brain activity in Veh-treated larvae (*p* > 0.05).

### 3.4. The Influence of PALM on c-fos and Bdnf Expression in PTZ-Treated Zebrafish Larvae

**Figure 4:**
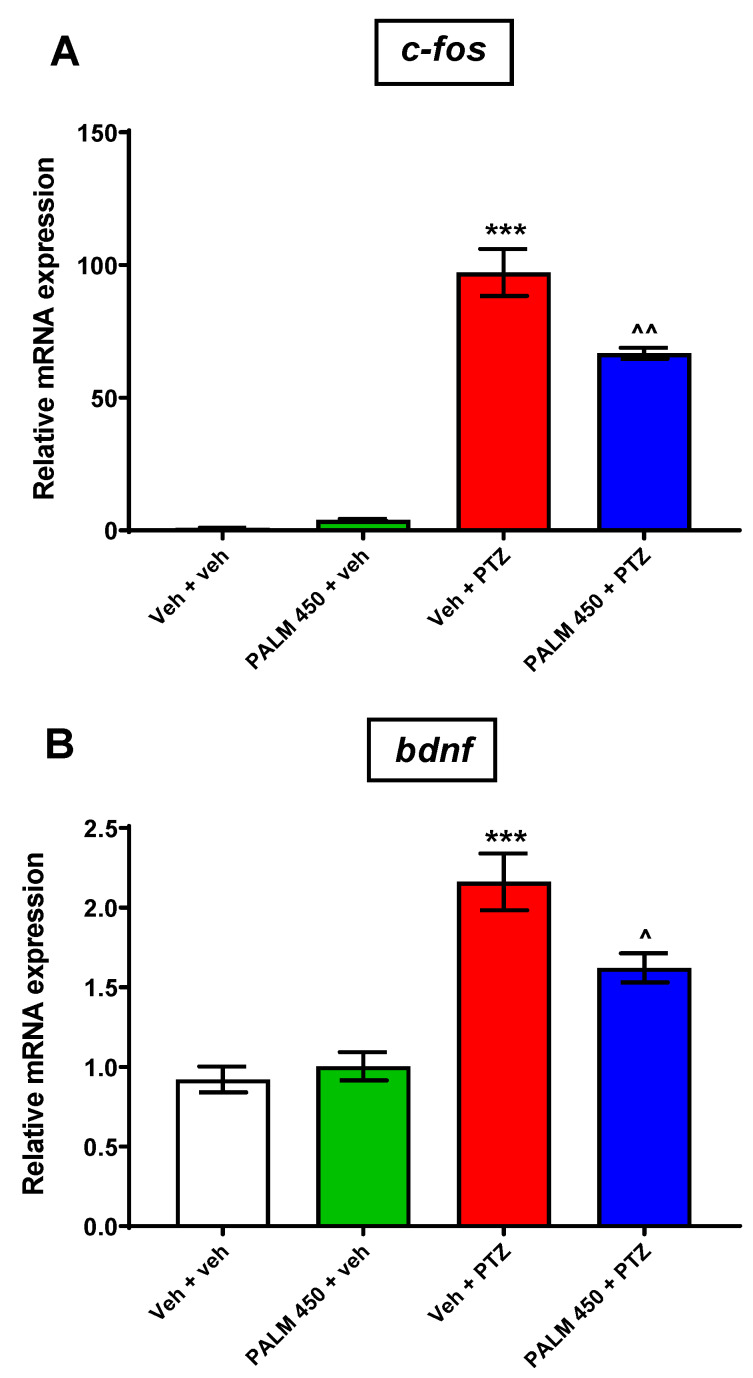
Evaluation of the effect of PALM (450 µM) on seizure-related markers expression in 7 dpf zebrafish larvae. PALM decreased PTZ-induced (**A**) *c-fos* and (**B**) *bdnf* upregulated expression. Data were analyzed using one-way ANOVA with the Tukey’s *post-hoc* test or Student’s *t*-test. Data are depicted as a mean ± SEM (*n* = 4/group, twice replicated). *** *p* < 0.001 vs. Veh + veh; ^^ *p* < 0.01, ^ *p* < 0.05 vs. Veh + PTZ. PALM—palmatine, PTZ—pentylenetetrazole, Veh—vehicle.

Since the anticonvulsant activity of BERB has been shown previously [22,23,24], here, we only assessed the effect of PALM on *c-fos* and *bdnf* expression in PTZ-treated zebrafish larvae. qRT-PCR expression analysis with one-way ANOVA revealed statistically significant differences in expression levels of *c-fos* (F (3, 28) = 109.0, *p* < 0.001) and *bdnf* (F (3, 28) = 24.64, *p* < 0.001; *n* = 4/group, twice replicated) between the tested groups of animals (Figure 4A,B). Tukey’s post-hoc test indicated that PTZ induced substantial increases in the expression of both *c-fos* and *bdnf* when compared to Veh-treated group (*p* < 0.001). Pretreatment with PALM (450 µM) significantly downregulated *c-fos* and *bdnf* expression induced by PTZ (*p* < 0.01 and *p* < 0.05, respectively). In the absence of PTZ, *c-fos* and *bdnf* expression in PALM (450 µM) + Veh-treated group was found to be similar to expression of both markers in Veh-treated group (*p* > 0.05) (Figure 4A,B).

### 3.5. The Influence of PALM or BERB on PTZ-Induced Hyperlocomotion in Zebrafish

**Figure 5:**
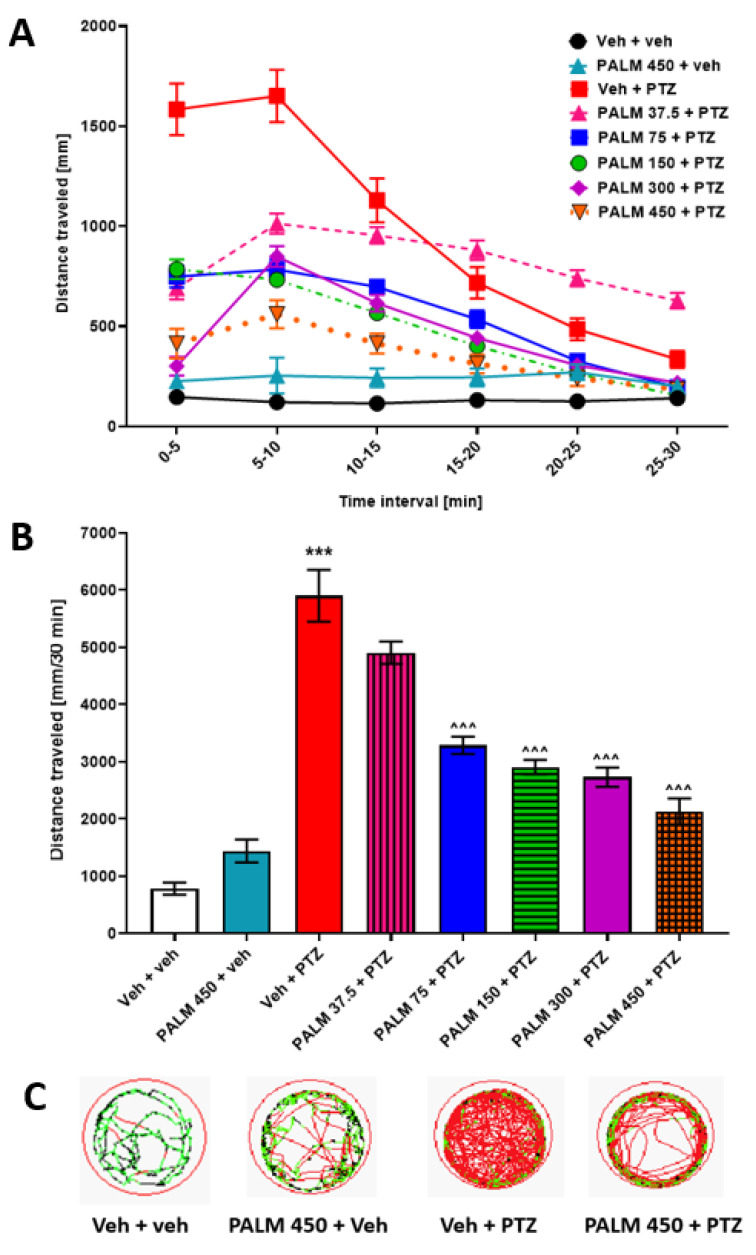
The influence of PALM on the seizure-like behavior in the PTZ-induced zebrafish hyperlocomotion assay. After a 24-h incubation in different doses of PALM (37.5, 75, 150, 300, or 450 µM), 7 dpf zebrafish larvae were exposed to 20 mM PTZ. Seizure-like behavior was assessed 5 min after acute PTZ exposure. Results of the assay are presented as (**A**) distance traveled in 5 min intervals, and (**B**) total distance traveled during 30 min of assay, (**C**) digital tracking map of larvae at second (5–10 min) interval of experiment: large movement—red, small movement—green. Data were analyzed using one-way or two-way with repeated measures ANOVA followed by the Tukey’s or Bonferroni’s *post-hoc* test, respectively. Data are depicted as a mean ± SEM. Veh + veh (*n* = 36), PALM 450 + veh (*n* = 26), Veh + PTZ (*n* = 34), PALM 37.5 + PTZ (*n* = 46), PALM 75 + PTZ (*n* = 48), PALM 150 + PTZ (*n* = 48), PALM 300 + PTZ (*n* = 47), PALM 450 + PTZ (*n* = 32). *** *p* < 0.001 vs. Veh + veh; ^^^ *p* < 0.001 vs. Veh + PTZ. PALM—palmatine, PTZ—pentylenetetrazole, Veh—vehicle.

As mentioned in the introduction section, PTZ-induced (20 mM) tonic-clonic-like seizures are correlated with hyperlocomotion in larval zebrafish. The effect of PALM in PTZ-induced hyperlocomotion assay was assessed at five concentrations (37.5, 75, 150, 300, 450 µM). Two-way ANOVA with repeated measures revealed statistically significant differences between tested groups of animals in different time intervals (group: F (7, 309) = 57.42, *p* ˂ 0.001; time: F (5, 805) = 182.7, *p* ˂ 0.001; group × time interaction: F (35, 1545) = 25.06, *p* ˂ 0.001; *n* = 26–48; Figure 5A). One-way ANOVA analysis of total distance indicated a concentration-dependent effect of PALM towards lowering of locomotor activity in PTZ-treated larvae, compared to PTZ-treated group (F (7, 309) = 57.42, *p* ˂ 0.001; *n* = 26–48; Figure 5B). Among the tested doses of PALM, the dose of 37.5 µM had a tendency to decrease locomotor activity of PTZ-treated larvae within the first 10 min of the experiment, but it did not decrease total locomotor activity of PTZ-treated larvae (*p* > 0.05) when compared to PTZ-treated animals. Thus, the dose of PALM 37.5 µM was chosen as inactive dose for further experiments. The highest concentration of PALM itself did not affect the locomotion of 7 dpf larval zebrafish (Figure 5A,B; for an example of traces, see Figure 5C).

**Figure 6:**
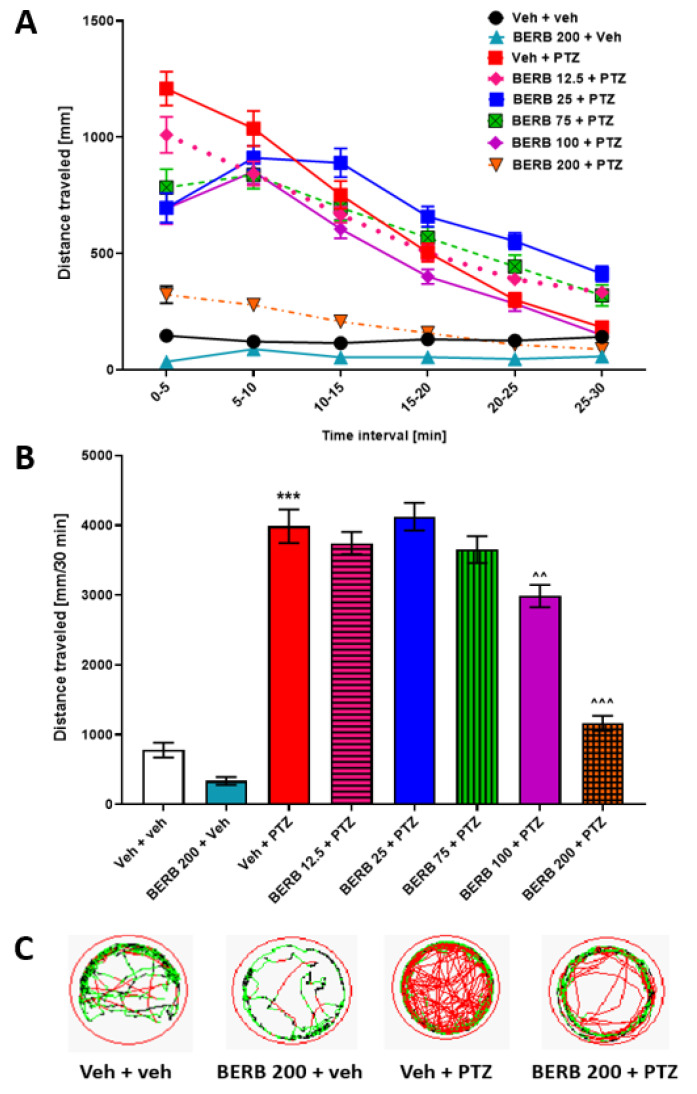
The influence of BERB on the seizure-like behavior in the PTZ-induced zebrafish hyperlocomotion assay. Seven dpf zebrafish larvae, after 24 h incubation with different doses of BERB (12.5, 25, 75, 100, or 200 µM), were exposed to 20 mM PTZ. Seizure-like behavior was assessed 5 min after acute PTZ exposure. Results of the assay are presented as (**A**) distance traveled in 5-min intervals, and (**B**) total distance traveled during 30 min of assay, (**C**) digital tracking map of larvae at second (5–10 min) interval of experiment: large movement—red, small movement—green. Data were analyzed using one-way or two-way with repeated measures ANOVA followed by the Tukey’s or Bonferroni’s *post-hoc* test, respectively. Data are depicted as a mean ± SEM. Veh + veh (*n* = 36), BERB 200 + veh (*n* = 29), Veh + PTZ (*n* = 48), BERB 12.5 + PTZ (*n* = 48), BERB 25 + PTZ (*n* = 48), BERB 75 + PTZ (*n* = 48), BERB 100 + PTZ (*n* = 48), BERB 200 + PTZ (*n* = 48). *** *p* < 0.001 vs. Veh + veh; ^^^ *p* < 0.001, ^^ *p* < 0.01 vs. Veh + PTZ. BERB—berberine, PTZ—pentylenetetrazole, Veh—vehicle.

Similarly, the effect of BERB in the PTZ-induced hyperlocomotion assay was assessed at five concentrations (12.5, 25, 75, 100, 200 µM). Two-way ANOVA with repeated measures revealed statistically significant differences between tested groups of animals in different time intervals (group: F (7, 345) = 73.13, *p* ˂ 0.001; time: F (5, 1103) = 165.4, *p* ˂ 0.001; group × time interaction: F (35, 1725) = 15.41, *p* ˂ 0.001; *n* = 29–48; Figure 6A). One-way ANOVA analysis of total distance also indicated the differences between tested groups of animals (F (7, 345) = 73.13, *p* ˂ 0.001; *n* = 29–48; Figure 6B). Among the tested doses of BERB, only the doses of 100 µM (*p* ˂ 0.01) and 200 (*p* ˂ 0.001) µM decreased locomotor activity of PTZ-treated larvae, compared to only PTZ-treated group. The 75 µM dose of BERB had a tendency to decrease the locomotor activity of PTZ-treated larvae within the first 5 min of the experiment, but it did not decrease total locomotor activity of PTZ-treated larvae (*p* > 0.05) when compared to PTZ-treated group. Therefore, the 75 µM dose of BERB was chosen as an inactive dose for further experiments. The highest concentration of BERB itself did not affect the locomotion of 7 dpf larvae (Figure 6A,B; for an example of traces see Figure 6C).

Based on the data obtained in the hyperlocomotion assay, the dose reducing PTZ-induced locomotor activity by 50% (ED_50_) was determined. PALM and BERB inhibited seizure-like behavior with ED_50_ value equal to 181.2 (141.6–231.7) µM, r = 0.92 and 138.5 (125.8–152.6) µM, r = 0.99, respectively (Figure S1). The log dose-probit linear regression analysis revealed the different slopes of regression curves of PALM and BERB to be −39.63 and −149.70, respectively.

### 3.6. The Influence of Extract of Berberis Sibirica Radix and Combination of PALM and BERB on PTZ-Induced Hyperlocomotion in Zebrafish

**Figure 7:**
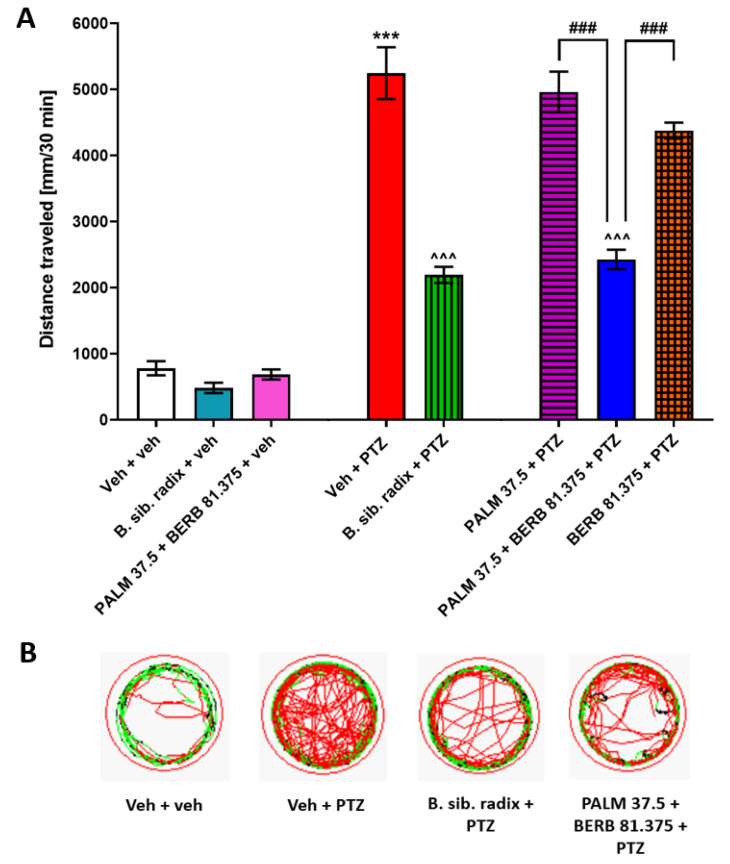
The influence of extract of *Berberis sibirica* radix and the combination of PALM and BERB in 1:2.17 ratio corresponding to the natural proportions of both alkaloids in the plant on the seizure-like behavior in the zebrafish PTZ-induced hyperlocomotion assay. Extract of *Berberis sibirica radix* (100 µg/mL) and alkaloids PALM (37.5 µM) and BERB (81.375 µM) alone or in combination were used. Seven dpf zebrafish larvae, after 24 h preincubation with investigated compounds alone or in combination, were exposed to 20 mM PTZ. Seizure-like behavior was assessed 5 min after acute PTZ exposure. Results of the assay are presented as (**A**) total distance traveled during 30 min of assay, and (**B**) digital tracking map of larvae at second (5–10 min) interval of experiment: large movement—red, small movement—green. Data were analyzed using one-way ANOVA, followed by the Tukey’s *post-hoc* test. Data are depicted as a mean ± SEM. Veh + veh (*n* = 36), B. sib. radix + veh (*n* = 24), PALM 37.5 + BERB 81.375 + veh (*n* = 32), Veh + PTZ (*n* = 45), B. sib. radix + PTZ (*n* = 47), PALM 37.5 + PTZ (*n* = 40), PALM 37.5 + BERB 81.375 + PTZ (*n* = 48), BERB 81.375 + PTZ (*n* = 32). *** *p* < 0.001 vs. Veh + veh; ^^^ *p* < 0.001 vs. Veh + PTZ; ### *p* < 0.001 vs. respective group. B. sib. radix—*Berberis sibirica radix*, BERB—berberine, PALM—palmatine, PTZ—pentylenetetrazole, Veh—vehicle.

The effects of the total methanolic extract of *Berberis sibirica radix* (100 µg/mL) and the combination of PALM and BERB in 1:2.17 ratio, corresponding to natural proportion of both alkaloids in the plant, were assessed in the PTZ-induced hyperlocomotion assay (Figure 7). Two-way ANOVA with repeated measures indicated statistically significant differences between tested groups of zebrafish in different time intervals (group: F (7, 296) = 76.22, *p* ˂ 0.001; time: F (5, 774) = 185.2, *p* ˂ 0.001; group × time interaction: F (35, 1470) = 26.41, *p* ˂ 0.001; *n* = 24–48; Figure S2). Additionally, one-way ANOVA analysis of total distance traveled indicated differences between groups of animals (F (7, 296) = 26.97, *p* ˂ 0.001; *n* = 32–48; Figure 7A; for an example of traces see Figure 7B). Tukey’s post-hoc indicated that combination of PALM and BERB in 1:2.17 ratio (37.5 µM and 81.375 µM, respectively) as found in *Berberis sibirica radix* methanolic extract significantly decreased hyperlocomotion of PTZ-treated larvae (*p* < 0.001) and their effect was similar to the effect of pure extract. The combination of PALM/BERB, or total *Berberis sibirica radix* extract did not affect the baseline locomotion of 7 dpf Veh-treated larval zebrafish (*p* > 0.05) (Figure 7A,B).

**Figure 8:**
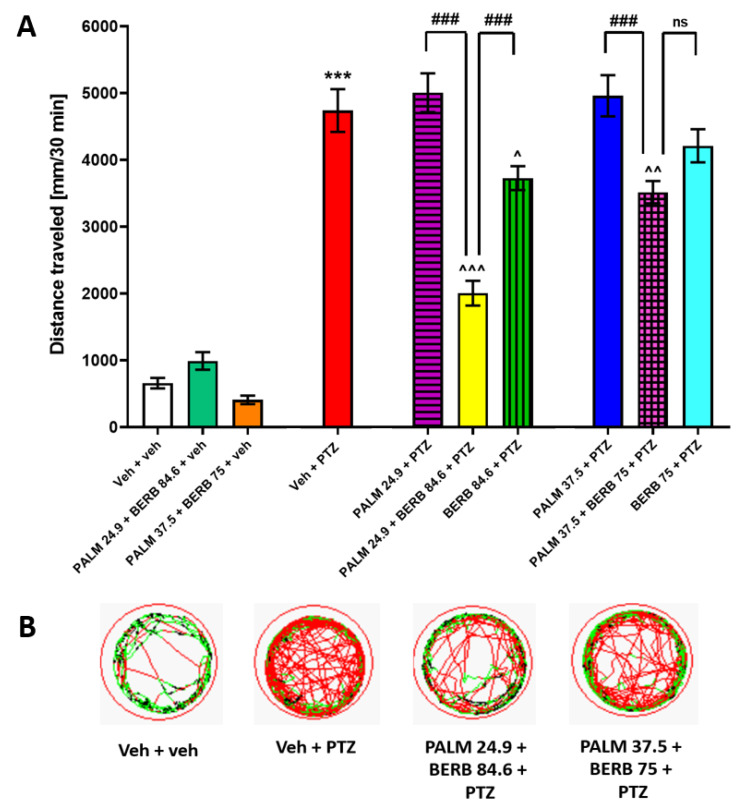
The influence of an artificially created combination of PALM and BERB on the seizure-like behavior in the zebrafish PTZ-induced hyperlocomotion assay. In the first set of experiments, alkaloids were used in doses equal to their ED_16_ values: PALM (24.9 µM), BERB (84.6 µM). In the second set of experiments, alkaloids were used at subeffective doses: PALM (37.5 µM) and BERB (75 µM). Zebrafish larvae after 24 h preincubation with investigated compounds alone or in combination were exposed to 20 mM PTZ. Seizure-like behavior was assessed 5 min after acute PTZ exposure. Results of the assay are presented as (**A**) total distance traveled during 30 min of assay, and (**B**) digital tracking map of larvae at second (5–10 min) interval of experiment: large movement—red, small movement—green. Data were analyzed using one-way ANOVA, followed by the Tukey’s *post-hoc* test. Data are depicted as a mean ± SEM. Veh + veh (*n* = 32), PALM 24.9 + BERB 84.6 + veh (*n* = 34), PALM 37.5 + BERB 75 + veh (*n* = 24), Veh + PTZ (*n* = 31), PALM 24.9 + PTZ (*n* = 31), PALM 24.9 + BERB 84.6 + PTZ (*n* = 39), BERB 84.6 + PTZ (*n* = 32), PALM 37.5 + PTZ (*n* = 39), PALM 37.5 + BERB 75 + PTZ (*n* = 48), BERB 75 + PTZ (*n* = 48). *** *p* < 0.001 vs. Veh + veh; ^^^ *p* < 0.001, ^^ *p* < 0.01, ^ *p* < 0.05 vs. Veh + PTZ; ### < 0.001 vs. respective group; ns—not statistically significant. BERB—berberine, PALM—palmatine, PTZ—pentylenetetrazole, Veh—vehicle.

The effect of two additional combinations of PALM and BERB was assessed in the PTZ-induced hyperlocomotion assay (Figure 8). The first set of experiments was performed using PALM, BERB and mixture of PALM and BERB in doses equal to the ED_16_ value of both compounds established in the experiment described above. In the second set of experiments, the investigated compounds were administered alone or in combination at subeffective doses established in the experiment described above.

Two-way ANOVA with repeated measures revealed statistically significant differences between the tested group of animals in different time intervals (group: F (9, 350) = 59.53, *p* ˂ 0.001; time: F (5, 951) = 161.90, *p* ˂ 0.001; group × time interaction: F (45, 1750) = 9.00, *p* ˂ 0.001; *n* = 24–48; Figure S3). One-way ANOVA analysis of total distance traveled indicated differences between groups of animals (F (9, 350) = 59.53, *p* ˂ 0.001; *n* = 24–48; Figure 8A; for example of traces, see Figure 8B). Tukey’s *post-hoc* showed that PALM and BERB in ED_16_ doses (24.9 µM and 84.6 µM, respectively) significantly decreased hyperlocomotion of PTZ-treated larvae, compared to PTZ-treated group (*p* < 0.001). Similarly, Tukey’s *post-hoc* showed that subeffective doses of PALM and BERB (37.5 µM and 75 µM, respectively) significantly decreased hyperlocomotion of PTZ-treated larvae, compared to PTZ-treated group (*p* < 0.01). The combination of alkaloids in their ED_16_ values and at inactive doses did not affect the locomotion of 7 dpf Veh-treated larvae (*p* > 0.05) (Figure 8A,B).

## 4. Discussion

To consider the applicability of PALM in the treatment of CNS disorders, mathematical modeling of its BBB penetration and QSAR studies were performed. In order to limit the differences between the actual and the estimated BBB values, the multiple linear regression (MLR) methodology with the backward elimination of variables [43] was used to create the QSAR model. In this study, the newly established QSAR model based on hydrogen bond acidity A values, the logarithm of *n*-octanol/water partition coefficient logP_o/w_ values, and the MW was utilized. Many attempts have been made to create adequate models, among others, the relationship between logBB and other Abraham descriptors [38], i.e., B, S, E, and V, has been investigated. Moreover, the relationship between logBB and topological polar surface area TPSA, polarizability and the difference between *n*-octanol/water and cyclohexane/water logP values (ΔlogP), which is related to the overall hydrogen-bonding ability of a molecule [44,45], was compared. In each of the above-mentioned estimations, weak values of statistical parameters, i.e., the adjusted sum of squares, adjusted mean square errors, standard errors, variance inflation factors, R^2^ values, *p*-values, T-values, and the Fisher criterion (F-values) parameters, were obtained, which indicates a poor fit of the models to real experimental data. Since the best fit was obtained by correlating logBB with A, logP_o/w_, and MW (Table 2), we assumed that the ability of alkaloids to cross the BBB is determined mainly by these parameters. Based on the analysis of variance of our QSAR model, it can be assumed that the proposed model will also be suitable for other isoquinoline alkaloids of plant origin from the subgroup of protoberberines. The limitations of the model have been examined by applying the applicability domain (AD) [46] based on the results presented in our previous paper [47]. The relationships between the standardized residuals and the leverages were investigated. The obtained results show that all substances are within the AD. Therefore, both the reliability and predictability of the model have been confirmed. Since PALM fits to this model, we concluded that theoretical estimations provided sufficient data to consider the effect exerted by PALM on the function of the CNS.

PALM investigated in this study was isolated from the methanolic extract of *Berberis sibirica* roots by a novel separation technique, CPC, which is operated in liquid–liquid conditions that help to isolate single molecules out of a rich matrix, such as plant extracts. Lack of solid support in the separation process enables a high recovery rate of the injected samples contrary to classical chromatography, where they can adsorb to the surface of a stationary phase [48]. So far, the technique has been widely studied and used for the isolation of single molecules of plant origin or for the enrichment of extracts [49]. In the scientific literature, several publications on the recovery of isoquinoline alkaloids have been published. High purity PALM was difficult to obtain, as in most of the cases, it was accompanied by a structurally similar BERB. This analytical problem was solved by prolonged separation methods, lower flow rates of solvents [25,50], high rotation speed values, or a two-step fractionation [51,52] that could increase the purity of the isolate. The methodology described above was applied here on HPCCC columns, on different instruments, which are characterized by two axes of rotation, contrary to CPC [48]. Finally, as the greatest advantage relative to CPC columns, we found the solvent system proposed by Tang et al. [25] resulted in the highest selectivity towards the metabolites present in Siberian barberry. Using this approach, PALM was obtained at a purity of 93%.

In the current study, we uncovered the anticonvulsant activity of PALM in the zebrafish larval PTZ-induced seizure assay. During the past decade, zebrafish have enjoyed widespread use in biomedicine and PTZ-induced seizure tests, both in adult and larval zebrafish, where they have been validated as valuable models to identify antiseizure compounds and mechanisms of epilepsy [7,9,10,53,54].

Previously, the anticonvulsant activity was confirmed for *Berberis integerrima* root extract in mouse [55] and for the main constituent of *Berberis* sp. extracts, BERB, both in rodents and zebrafish [22,23,24]. However, although CNS-related effects of PALM have been investigated [56,57,58,59,60], until now, no evidence has existed for its activity in animal seizure and epilepsy models. Here, we demonstrated for the first time the anticonvulsant activity of PALM, which decreased the number of epileptiform-like discharges recorded in the brain of PTZ-treated zebrafish larvae. This phenomenon was accompanied by a reduction of PTZ-induced hyperlocomotion, which is considered as a behavioral measure of seizure activity. The effect was clearly dose-dependent, with an ED_50_ value of 181.2 μM. Moreover, our study confirmed the anticonvulsant effects of BERB in larval zebrafish with an ED_50_ value of 138.5 μM. Although the range of effective doses are similar, the slope of the dose–response curve for both compounds is different, which implies that the mechanism of anticonvulsant activity of PALM and BERB may be different. This assumption seems to be justified by the finding that combinations of PALM and BERB at subthreshold doses resulted in a clear-cut anticonvulsant action, and at doses corresponding to their ED_16_ values, anticonvulsant action even exceeded 50%, thus suggesting hyperadditive drug interaction. To further explore this issue, we assessed the effect of both the authentic extract of *Berberis sibirica radix* and an artificially composed mixture of PALM and BERB at a proportion of 1:2.17, which corresponds to the content of both compounds in the natural extract of *Berberis sibirica radix*. We discovered that both the natural extract and the mixture of PALM and BERB possess anticonvulsant properties. These results suggest that coexistence and interaction of PALM and BERB is responsible for the anticonvulsive activity of *Berberis* sp. extract.

The detailed mechanism of anticonvulsant action of BERB and PALM is unknown, but we hypothesize that it might result from its GABAmimetic activity. The proconvulsant effect of PTZ in the zebrafish larvae is related to the attenuation of GABAergic neurotransmission—both through GABA_A_ receptor antagonism and decrease in GABA level [61]. The anticonvulsant activity of numerous GABAmimetic compounds, including some clinically used ASDs, has been previously proved in the zebrafish larvae PTZ seizure assay [10,62,63]. However, this mechanism of anticonvulsant effect of the studied alkaloids might be also questioned because it was demonstrated that PALM did not change the GABA content in the mouse brain [59], and BERB was able to inhibit the GABA_A_ receptor at approximately 60% [64].

Previous studies evaluating the effects of PALM on behavior and CNS neurotransmission revealed several possible mechanisms of its action. An enhancement of learning and memory processes by PALM in mice was reported and the inhibition of acetylcholinesterase (AChE) activity in the brain was proposed as a mechanism of action [57]. The engagement of the GABA-benzodiazepine pathway in this regard was also considered [57]. On the other hand, sedative and hypnotic effects of PALM were linked to changes in concentration of neurotransmitters, i.e., dopamine and serotonin, in brain structures [58,59]. It was found that PALM exerted antidepressant activity in the tail suspension, sucrose preference, and forced swim tests both in stressed and unstressed mice. It was suggested that this result was due to the inhibition of monoamine oxidase A (MAO-A) and catalase activity, as well as a decrease in plasma nitrite and corticosterone levels in the brain observed after PALM administration [56]. MAO-A and MAO-B are mitochondrial enzymes that inactivate monoaminergic neurotransmitters after their synaptic reuptake. Although studies confirm contribution of monoamines in epileptogenesis as well as seizure generation and propagation processes, specifying their role is difficult due to complexity of neurotransmission. The anticonvulsant properties of the selective MAO-A inhibitor, i.e., LU43829 (esuprone), in the electrical kindling model of epilepsy in rats were reported previously by Löscher et al. [65]. MAO-A knockout mice had increased susceptibility to electrically- and PTZ-induced seizure activity, but were more resistant to epileptogenic processes [66]. Considering previous reports on inhibition of MAO-A activity by PALM [56], it might be assumed that decreased hyperlocomotion in the zebrafish larvae exposed to PTZ might result from interaction with this enzyme. A single MAO form was identified both in adult [67] and larval (42 h post-fertilization [68]) zebrafish and is more similar to mammalian MAO-A than to MAO-B. The zebrafish MAO displays 70% identity with human and rat MAO forms and possesses the same substrate binding sites that were identified in MAO-A [67]. Deprenyl (MAO inhibitor) exposure significantly increased serotonin content in the brain of developing larval zebrafish and decreased their locomotion and altered vertical positioning [68]. PALM-induced reduction of locomotor activity was previously noted in mice [59]. In the present study, we did not observe changes of locomotor activity in PALM-only-treated zebrafish larvae, which could be due to relatively low ability of PALM to elicit MAO inhibition and/or participation of other mediators, i.e., tryptophan hydroxylase, that regulate serotonin level in the brain [68]. Thus, it can be concluded that decrease of PTZ-induced hyperlocomotion observed in our study did not result from MAO inhibition by PALM in zebrafish larvae.

Since none of the previously reported (suggested) mechanisms involving alterations in neurotransmission may explain the antiseizure activity of PALM in the PTZ-induced seizure assay in zebrafish, we estimated the expression of *c-fos* and *bdnf* genes—a gold standard for detecting synaptic activity which participates in seizure generation [9], to verify if PALM-induced changes in EEG measurements in PTZ-treated zebrafish larvae were correlated to intracellular changes. *c-fos* is an immediate response gene induced within minutes by neuronal activity, and is a key player in regulating neuronal cell survival and death [69]. c-fos regulates expression of BDNF, a neurotrophic factor, which plays important roles in neurogenesis, neuronal maturation, and synaptic plasticity [70]. Results of our study demonstrated that EEG and behavioral events correlate with molecular measurements, as PALM decreased both the number of epileptiform-like discharges in EEG and hyperlocomotion in the PTZ-treated group, as well as lowered *c-fos* and *bdnf* mRNA expression. Moreover, this observation is in agreement with previous studies that showed that compounds with anticonvulsant properties might reduce both *c-fos* and *bdnf* expression [20,71].

Interestingly, PALM was found to diminish allodynia, hyperalgesia, and depressive behavior in rats with concomitant diabetic neuropathic pain and depression. These effects were discussed in relation to a decrease in the P2X7 receptor expression and phosphorylation of extracellular signal-regulated kinase 1 and 2 (ERK 1/2) in the hippocampus [72]. Importantly, data obtained both from experimental models of epilepsy [73,74,75,76] and epileptic patients [75,76] revealed increased expression of P2X7 receptors in brain structures during and after seizure cessation. Blockers of P2X7 receptors, i.e., Brilliant Blue G, AFC-5128, JNJ-47965567, tanshinone IIA sulfonate, and A-438079, provided moderate to meaningful antiseizure effect in the experimental models of epilepsy [75,77,78], although they showed only modest anticonvulsant effects on acute convulsions in rodents [77,78]. The ERK 1/2 signaling pathway is involved in the regulation of synaptic plasticity and neuronal excitability and might, therefore, contribute to both seizure generation and propagation [79,80]. Previous studies showed that ERK 1/2 activation accompanies 4-aminiopiridine- [81], kainic acid- [82], PTZ- [83], and pilocarpine-induced seizures [84,85,86] as well as audiogenic seizures [87]. Significantly higher levels of phosphorylated ERK 1/2 were also detected in the temporal lobe of patients with intractable epilepsy [88]. Moreover, inhibition of the ERK 1/2 pathway prevented audiogenic seizures in rats [87], and ERK 1/2 is postulated as a potential target in future strategy of epilepsy treatment [84,87,89].

Notably, Krens et al. [90] reported that both ERK 1 and ERK 2 are present in the zebrafish genome. Admittedly, neither contribution of P2X7 receptors nor ERK 1/2 activity in the zebrafish PTZ-induced seizure test have yet been investigated. However, the high percentage of homology of zebrafish and human genomes (approximately 70% homology, with 84% of the genes associated with human diseases having an equivalent in the zebrafish genome [91]) suggests strong resemblance between these signaling pathways. Moreover, previous studies have proved the similarity between zebrafish and other vertebrate brains, including rodents and human, with respect to structural organization and neurochemistry. Importantly, zebrafish possess the same neurotransmitters and components of neurotransmission, i.e., enzymes, neurotransmitter receptors or transporters that regulate synthesis and metabolism of neurotransmitters, as mammals [92]. Additional confirmation of the similarity between zebrafish and mammals might also be attributed to the high reproducibility of results obtained in the zebrafish and rodent models of seizures [11,12,53,93].

## 5. Conclusions

In conclusion, our study revealed, for the first time, the anticonvulsant activity of PALM. Moreover, our results suggest that BERB and PALM may exhibit antiseizure activity through different mechanisms. From a therapeutic standpoint, the combination of PALM and BERB appears to be more favorable than usage of both compounds separately.

## Figures and Tables

**Table 1:** Physicochemical parameters (ACD/Percepta software) and the experimentally obtained logBB values (logBB_exp_) [30,31,32,33,34,35,36,37].

Name	A	logP_o/w_	MW	logBB_exp._
Tetrahydropalmatine	0	3.373	355.43	0.43
Tetrahydroberberine	0	3.084	339.39	0.39
Coptisine	0	4.193	320.32	0.46
Isorhynchophylline	0.41	3.304	384.47	−0.59
Rhynchophylline	0.41	3.304	384.47	−0.59
10-dehydroxyl-12-demethoxy-conophylline	0.5	7.675	794.89	−0.92
Camptothecin	0.17	0.742	384.35	−0.29
Topotecan	0.67	0.962	421.45	−0.42
Nuciferine	0	4.107	295.38	−0.52
Nornuciferine	0.13	3.752	295.38	−0.18
Berberine	0	4.499	336.36	−0.17
Huperzine a	0.47	1.483	242.32	−0.70
Dehydrocorydaline	0	5.405	366.43	−0.59

**Table 2:** Analysis of variance obtained for Equation (1).

Descriptors	SE Coefficient	*p*-Value	Variance Inflation Factors (VIF)	F-Value
A	0.670	0.003	3.67	18.82
logP_o/w_	0.125	0.016	3.32	10.06
MW	0.00195	0.096	1.26	3.71

## Supplementary Materials

The following are available online at https://www.mdpi.com/2073-4409/9/5/1233/s1, Figure S1: Effect of PALM and BERB on the PTZ-induced seizure-like behavior in the zebrafish hyperlocomotion assay – the log dose-probit linear regression analysis, Figure S2: The influence of extract of *Berberis sibirica radix* and the combination of PALM and BERB in 1: 2.17 ratio corresponding to the naturally occurring proportion of both alkaloids in the plant on seizure-like behavior in the zebrafish PTZ-induced hyperlocomotion assay, Figure S3: The influence of an artificially created combination of PALM and BERB on seizure-like behavior in the zebrafish PTZ-induced hyperlocomotion assay.
