# Supplementary material for: The Influence of Palmatine Isolated from Berberis sibirica Radix on Pentylenetetrazole-Induced Seizures in Zebrafish
Source: Cells. 2020 May 16;9(5):1233. doi: 10.3390/cells9051233 (PMC7290958; doi:10.3390/cells9051233)
Supplement: Supplementary file 1 [file cells-09-01233-s001.pdf]

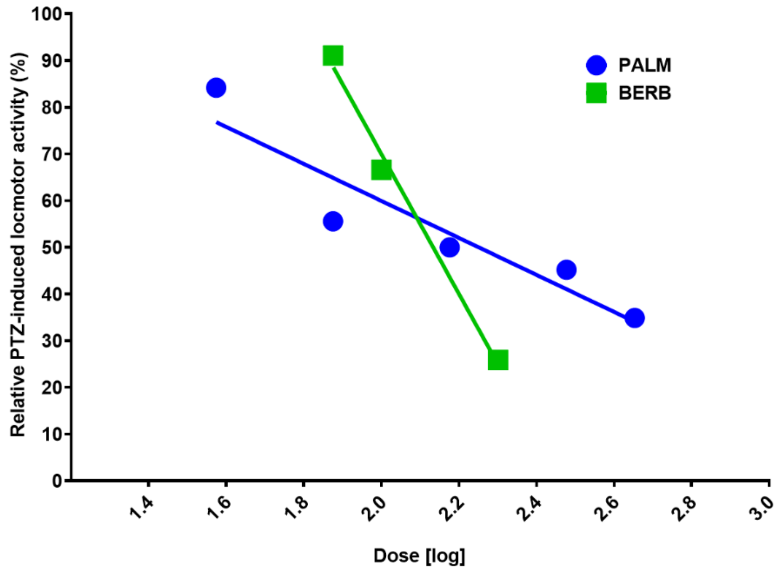

Figure 1. Effect of PALM and BERB on the PTZ-induced seizure-like behavior in the zebrafish hyperlocomotion assay – the log dose-probit linear regression analysis. Probit-log dose regression curves were calculated using Graph-PAD software: PALM y = -39.63x + 139.25, r = 0.88; BERB y = -149.70x + 369.4, r = 0.99.

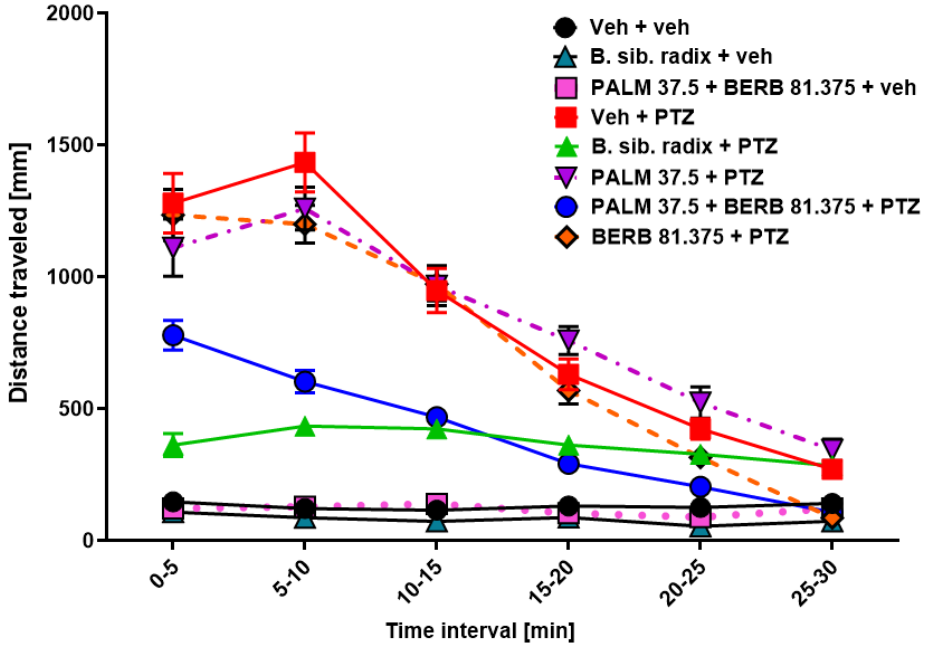

Figure 2. The influence of extract of *Berberis sibirica radix* and the combination of PALM and BERB in 1: 2.17 ratio corresponding to the naturally occurring proportion of both alkaloids in the plant on seizure-like behavior in the zebrafish PTZ-induced hyperlocomotion assay. Extract of *Berberis sibirica radix* (100 µg/ml), alkaloids PALM (37.5 µM) and BERB (81 µM), alone or in combination, were used. Zebrafish larvae after a 24 h long pre-incubation with tested compounds alone or in combination were exposed to 20 mM PTZ. Results of the assay are presented as distance traveled in 5 min intervals. Data were analyzed using two-way ANOVA with repeated measures, followed by Bonferroni's *post-hoc* test. Data are depicted as the mean ±SEM. Veh + veh (n=36), B. sib. radix + veh (n=24), PALM 37.5 + BERB 81.375 + veh (n=32), Veh + PTZ (n=45), B. sib. radix + PTZ (n=47), PALM 37.5 + PTZ (n=40), PALM 37.5 + BERB 81.375 + PTZ (n=48), BERB 81.375 + PTZ (n=32). BERB – berberine, PALM – palmatine, PTZ – pentylenetetrazole, Veh – vehicle.

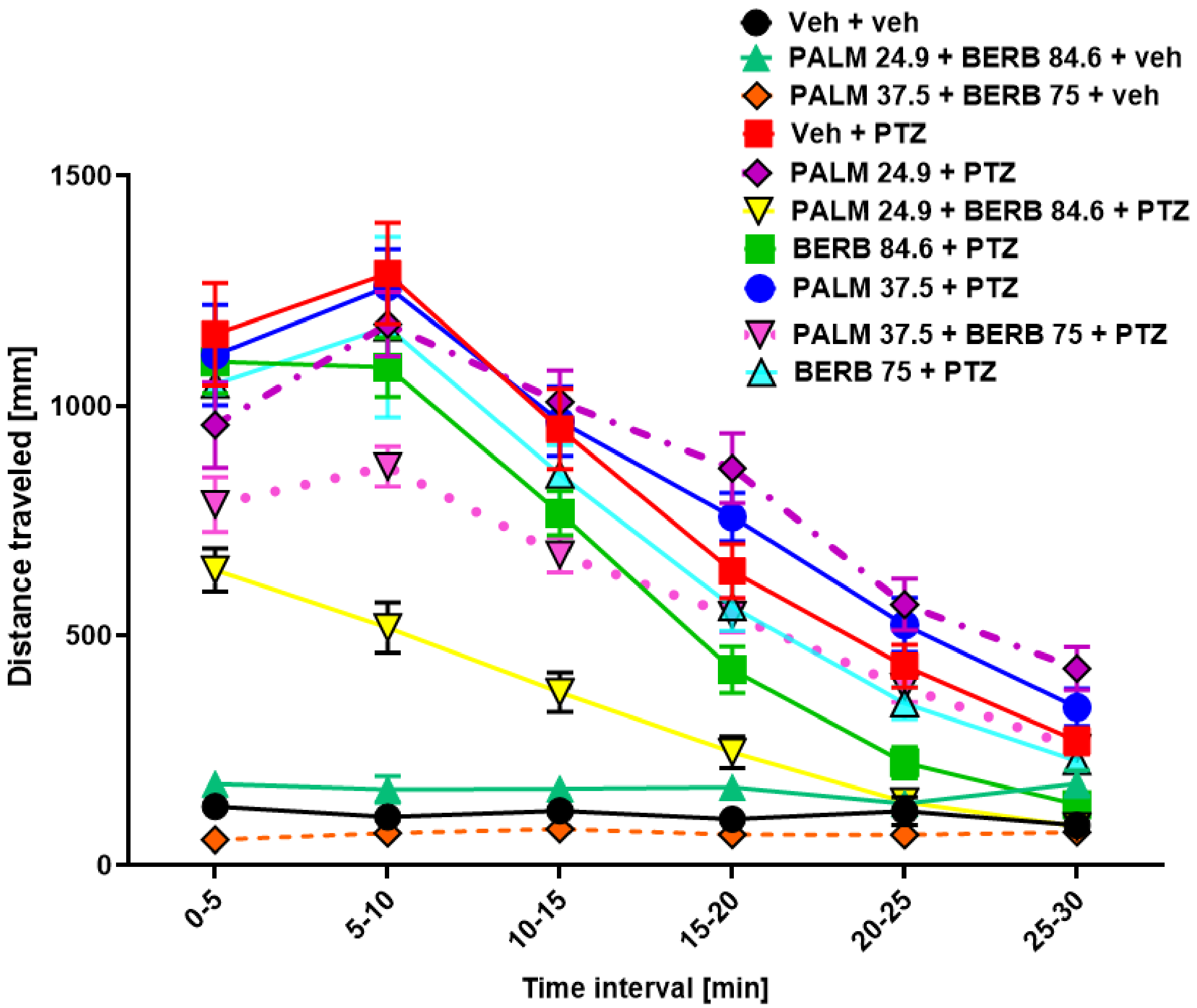

Figure 3. The influence of an artificially created combination of PALM and BERB on seizure-like behavior in the zebrafish PTZ-induced hyperlocomotion assay. In the first set of experiments, alkaloids were used in doses equal to their ED_{16} values, PALM (24.9 µM), BERB (84.6 µM). In the second set of experiments, alkaloids were used at sub-effective doses, PALM (37.5 µM) and BERB (75 µM). Zebrafish larvae after 24 h long pre-incubation with investigated compounds alone or in combination were exposed to 20 mM PTZ. Seizure-like behavior was assessed 5 min after acute PTZ exposure. Seizure-like behavior was measured 5 min after acute PTZ exposure. Results of the assay are presented as distance traveled in 5-min intervals. Data were analyzed using two-way ANOVA with repeated measures, followed by Bonferroni's *post-hoc* test. Data are depicted as a mean ± SEM. Veh + veh (n=32), PALM 24.9 + BERB 84.6 + veh (n=34), PALM 37.5 + BERB 75 + veh (n=24), Veh + PTZ (n=31), PALM 24.9 + PTZ (n=31), PALM 24.9 + BERB 84.6 + PTZ (n=39), BERB 84.6 + PTZ (n=32), PALM 37.5 + PTZ (n=39), PALM 37.5 + BERB 75 + PTZ (n=48), BERB 75 + PTZ (n=48). BERB – berberine, PALM – palmatine, PTZ – pentylenetetrazole, Veh – vehicle.
